# The role of environmental tax on the environmental quality in EU counties: evidence from panel vector autoregression approach

**DOI:** 10.1007/s11356-024-33632-z

**Published:** 2024-05-14

**Authors:** Buket Savranlar, Seyyid Ali Ertas, Alper Aslan

**Affiliations:** 1 Vocational School, Accounting and Tax Applications, Nisantaşi University, Istanbul, Turkey; 2 Yozgat Vocational School, Department of Property Protection and Security, Social Security Program, Yozgat Bozok University, Yozgat, Turkey; 3 Faculty of Aeronautics and Astronautics, Department of Aviation Management, Erciyes University, Kayseri, Turkey

**Keywords:** Environmental tax, CO_2_ emissions, EKC hypothesis, European Union, PVAR, *H23*, *Q53*, *C23*

## Abstract

This study intends to analyze the influence of environmental taxes on pollution in EU-27 nations. Furthermore, energy from renewable sources consumption and urbanization are employed to clarify CO_2_ emissions in this study that tests the EKC hypothesis. According to the findings, an increase in environmental taxes reduces CO_2_ emissions by 0.14%. Also, the data supported the validity of the EKC concept. The findings of the causality test demonstrated that there is a bidirectional causal link between CO_2_ emissions and environmental taxes. These results reflect that environmental tax revenues contribute to sustainability as an effective policy tool in EU countries. Policies regarding environmental tax enforcement come to the fore in terms of both keeping the balance in economic activities and serving sustainability.

## Introduction

Environmental issues such as global warming with air pollution, global climate change, and biodiversity reduction are within the main scope and impact of the economy. The quality of the air and the protection of the environment, which are environmental elements, are an important public commodity (Theeuwes 1991:67–68; He et al. 2018:7456). Reducing air pollution and therefore improving the quality of air can be treated as a national public commodity with no competition in its consumption and inability to exclude it. Because of this dimension of publicity, countries have various responsibilities in solving environmental problems and improving environmental quality. Accordingly, countries develop policies to ensure and sustain the environment, while also having a regulatory function through regulations, especially in the elimination of environmental negative externalities. All around the world, air pollution has been linked to health issues for people (Lelieveld et al. 2015; Cohen et al. 2017; Heft-Neal et al. 2018). PM_2:5_, which might permeate profoundly entering the bloodstream and respiratory system, leading to illnesses (Lelieveld c., 2015; Li et al. 2019), is the principal source of air pollution. Climate change is the most serious environmental issue that humanity has ever faced Change I P O C (2001). The earth’s surface temperature has been rising over the past 30 years. The dangers of major detrimental consequences on human life, property, the economy, and the environment have considerably grown as global warming rates and amplitudes continue to rise.

The major component of greenhouse gas emissions is CO2. CO2 emissions from economic activities, particularly conventional patterns of energy use based mostly on non-renewable, have become the principal human driver driving global warming from a global viewpoint (Meinshausen et al. 2009; Sari and Soytas 2009). When compared to 2005, worldwide CO2 emissions increased by around 5 109 tons in 2015. CO2 emissions in wealthy nations decreased by 1:1 109 tons, whereas those in developing nations climbed by 6:1 109 tons, essentially canceling out the developed countries’ emission reduction efforts (BP 2016). Developing nations emit enormous amounts of CO2 because of their aggressive promotion of industrialization and urbanization. Developing countries would endure more severe environmental damage and climate change repercussions than developed countries. More than 170 nations agreed to the Paris Agreement in 2016, pledging to work toward a long-term objective of keeping global warming to 2 degrees Celsius or less. It is critical to examine variations in global Carbon emissions to achieve this aim. Environmental Two factors determine the quality of the air: first, the direct output of human production activities, which are the primary causes of pollution and include economic expansion (Li et al. 2019), industrialization development (Gan et al. 2018), and energy use (Khan et al. 2019). In addition to this, excessive emissions and inadequate environmental spending will result in a decline in air quality.

At this point, the effects of regulating environmental stewardship are investigated using a variety of methodologies (Laplante and Ristone 1996) emphasizing three distinct perspectives: (1) Blackman and Kildegaard (2010) investigate environmental agency safety checks in Mexico and obtain that regulatory stress is unrelated to reducing pollution; According to (2) Lanoie et al. (2011), regulations will make it more expensive for companies to minimize pollution and release, drive away valuable resources, and lower efficiency and market competitiveness, making environmental issues unmanageable. By global cooperation, agreements for the solution of environmental problems have been raised, the negative effects and negative effects of environmental pollution have become a concern in the social sphere, the causes and consequences of pollution have been the focus of research in academic circles, and ways to improve the quality of the air have been discussed in the prevention and reduction of pollution.

Only a few academics have highlighted concerns regarding environmental taxes. Using the variance (DID) approach, Lin and Li (2011) studied the effects of carbon pricing on governance systems in five Northern countries. Many studies investigated the various aspects of a carbon tax. Lin and Li (2011) discovered that while the carbon price lowered CO_2_ emissions substantially in Finland, the effects were significantly negative in Denmark and Sweden. The majority of academics still think that environmental levies improve environmental governance. González and Hosoda (2016) used a Bayesian structure time series model to assess the impact of an aviation oil fuel tax on national transportation consumption growth in Japan, and they discovered that the tax decreased Emissions of CO_2_ by planes. In this context, agreements for solving environmental problems have been raised by global cooperation, the negative effects and negatives created by environmental pollution in the social sphere have become a concern, and in academic circles, the causes and consequences of pollution have been the subject of research and ways to prevent and reduce pollution have been discussed.

In keeping with the emissions and sustainable development targets, there has been a discernible growth in environmental pollution taxes inside the European Union (EU). The goal of taxation is to reduce carbon emissions to a manageable 5%. Energy, environment, and transportation taxes are among the taxes imposed for this purpose, particularly in Slovenia, Poland, France, Portugal, Finland, Latvia, Ireland, and Denmark. The EU is taking what are arguably the most obvious actions in the world in this regard. To lessen the detrimental externalities that third parties produce in production and consumption, the EU has imposed emission and environmental fees. Pollution, land degradation, and the greenhouse effect which raises living standards, lowers product quality, lowers income, and consumes more energy are examples of negative externalities.

More work is needed to regulate the release of toxic compounds into the atmosphere to safeguard the sustainability of ecosystem services, and the well-being of European populations, and prevent hazardous disruption of the global climate system. Numerous strategies are theoretically possible to further lower pollution in the future. For instance, one of its main objectives might be to lower environmental pollution and raise air quality through the usage of environmental levies.

Ecological taxation aims to transfer the tax burden from economically desirable social goods, such as jobs, income, and investments, to economically undesirable social goods, such as waste and environmental damage (Bosquet 2000). In addition, environmental taxes have nearly also been set at a lower level than what is warranted by environmental damages in Europe and have instead been utilized to raise income. Is it reasonable to assess the environmental impact and economic efficiency of a tool whose primary objective is to generate revenue? Secondly, environmental taxes are not self-contained. In Europe, they are frequently used to supplement existing rules with standards and guidelines, and they are frequently utilized to accelerate the adoption of new technology. A fundamental challenge is separating the effects of environmental taxes from other forms of regulation that are in force at the same time.

Unlike previous research, this study has looked at how environmental taxes affect EU carbon emissions. The impact of urbanization and the use of renewable energy on emissions of carbon are investigated for this reason. To the best of our knowledge, this study used the panel VAR technique to investigate, for the first time, the impact of energy use, urbanization, and environmental levies on the quality of the environment for EU nations. Our analysis also contributes to the econometric structure. We must enforce the requirement that the fundamental framework be the same for every cross-section unit when using the VAR process on panel data. One method to get around the parameter limitation is to introduce the fixed effects that are depicted in the model to make room for “individual variability” in the variable values, as this restriction is likely to be broken in practice. The delays of the variables that are dependent link fixed effects to regressors; therefore, biased coefficients will result from the standard averaging approach used to remove fixed effects. We employ a forward mean difference, also known as the “Helmert procedure,” to get around this issue. Only the forward average—that is, the average of all future data that are accessible for each nation year—is eliminated by this process. We may employ lagged regression coefficients as tools and estimate the coefficients using the system GMM since this transformation maintains the orthogonality between the modified variables and the regressors (Love and Zicchino 2006).

The remaining research is organized as follows: The relevant empirical literature is presented in the “Literature review” section. The “Model specification, data and methodology” section explains the technique, model, and data. Empirical results are included in the “Empirical findings” section. Concluding thoughts and policy implications are presented in the “Conclusion” section.

## Literature review

Scholars, policymakers, and economists have been debating the connections between energy usage, environmental quality, and taxes connected to the environment for the past thirty years. The single-country and multi-country data analysis situations covered in this literature review have received the majority of attention in the research that is currently accessible. After reviewing the literature, we can organize it into three broad categories since research has been done on topics like environmental taxes, consumption of energy and the economy, and the relationship between energy use and the environment.

Very recently, Bekun (2024) has examined the relationship between conventional energy use, agricultural practices, economic growth, and environmental sustainability in South Africa by using Pesaran’s Autoregressive distributed lag (ARDL) method, as well as the dynamic ARDL simulations method. To meet the study’s objectives, a carbon income function is fitted to annual frequency data from 1975 to 2020. Bekun observed that economic expansion, fossil fuel energy use, and agricultural activities all have a negative impact on environmental sustainability in South Africa, implying a trade-off between economic growth and environmental quality. Bekun (2022) studied how renewable and non-renewable energy, economic growth, and energy sector investment affect CO2 emissions in India. The long-run elasticity of the variables was determined using canonical cointegration regression (CCR), completely modified least squares (FMOLS), and dynamic least squares (DOLS). Granger causality analysis was employed to determine the direction of the causal relationship between the variables that were highlighted. The results of empirical regression indicate a negative correlation between renewable energy and CO_2_ emissions. The long-run elasticity of the variables was determined using canonical cointegration regression (CCR), completely modified least squares (FMOLS), and dynamic least squares (DOLS), and the direction of the causal relationship between the highlighted variables was determined using Granger causality analysis. The results of empirical regression indicate a negative correlation between renewable energy and CO_2_ emissions. Nadiri et al. (2024) identified carbon dioxide (CO2) emissions as the main cause of the urgent problem of environmental deterioration, endangering the sustainability of the environment worldwide, particularly the member states of the European Union (EU). The findings demonstrate that while globalization, eco-innovation, carbon taxes, and renewable energy all help to slow down environmental degradation, economic development also helps to lessen the problems associated with environmental sustainability in EU member states. Xu et al. (2023) gathered information from 287 cities between 2010 and 2019 to examine the mechanism behind the fee and determine its effect on lowering pollution. The findings indicate that the environmental tax had a major knock-on effect on sewage, waste gas, and solid waste emissions. This suggests that intergovernmental cooperation and regional collaboration can improve the implementation of environmental tax policies and their emission reduction effects. Dmytrenko et al. (2024) investigated the effects of environmental levies and stricter environmental regulations on greenhouse gas emissions in a sample of eight European nations from Central and Western Europe. Our findings indicate that only in Western Europe does the strictness of environmental regulations have a major impact. It is interesting to note that R&D spending ended up having the biggest impact on both groups. Guo and Wang (2018) used annual time series data from 1985 to 2014 to investigate the connection between Beijing, China’s carbon emissions and environmental regulations. The authors experimentally investigated the consequences of environmental legislation in Beijing using the Johansen Cointegration, VAR model, and impulsive reaction function methodologies. Environmental restrictions, according to the research, could help to foster technical development while also reducing the effects of carbon emissions. with relation to energy, the environment, the economy, and economic competitiveness. In a similar vein, Wolde-Rufael and Mulat-Weldemeskel (2022) discovered that environmental taxes and CO2 emissions in Latin American and Caribbean nations were negatively correlated. A similar conclusion was obtained about environmental taxes by Safi et al. (2021) after analyzing the impact of R&D and environmental taxes on carbon emissions in G7 nations. The impact of environmental tax measures on developing, developed, and growing nations was studied by Cottrell et al. (2015). Environmental fiscal policies that stray from the ideal tax design, according to the research, help to avert negative implications for international competition. The research argues that environment tax reform is a viable and affordable policy instrument for both long-term environmental conservation and economic prosperity. Morley (2012) examined the long-term impact of ecological tariffs on energy usage and pollutant emissions using data from 25 European nations between 1995 and 2005. The two-step GMM technique was used in this study to account for variability and unobserved heterogeneity difficulties in econometric assumptions. Environmental levies do not have a major impact on energy usage in the nations analyzed, according to the findings of the empirical research. Wang et al. (2015) used data from 36 Chinese industries to investigate the effects of carbon prices on industrial competitiveness. The authors conducted a thorough examination of the impact of carbon-related levies on various economic sectors. Fremstad and Paul (2019) looked at how carbon prices affect income disparity and general economic development in the United States. The authors came to the conclusion that the labor tax cuts associated with the carbon tax are being reduced, which is not enough to preserve Americans’ purchasing power. Peng et al. (2019) looked at how energy taxes might affect Jiangsu’s potential for prosperity and economy. Empirical research indicates that while energy taxes are advantageous for conserving resources and lowering energy usage, they also compromise economic and welfare goals. Rogan et al. (2011) investigated the impact of targeted policies on vehicle purchasing trends in the direction of lowering carbon-emitting automobiles. Drawing on statistics from Ireland, the authors concluded that at the start of a new taxing scheme, new-vehicle carbon emissions might be decreased by up to 13%. Ciaschini et al. (2012) assessed the relationship between taxes on the environment and carbon emissions from Italy using yearly time series data spanning from 2004 to 2007. The authors used an empirical general equilibrium technique (CGE) to hypothesize that tax policies might have an impact on regional prices, employment rates, economic growth, and carbon dioxide emissions. Miller and Vela (2013) investigated how environmental levies affected the reduction of pollutant emissions in fifty distinct nations. To test the key hypothesis, the researchers gathered annual data from 1995 to 2010 and used pass dynamics regression analysis. Stern et al. (1993) established a popular theoretical framework to explain pro-environmental behavior. They propose a social–psychology paradigm in which egoistic, social–altruistic, and biocentric value perspectives can all motivate impact on the environment. They next put the model to the test utilizing survey results. While they find widespread agreement for their approach, they also discover that only self-interested reasons are a true indicator of willingness to pay through taxes. The implicit pollution tax in the US quadrupled between 1990 and 2008, resulting in a 60% decrease in air pollutants from manufacturing industries despite a notable increase in industrial production (Shapiro and Walker 2018). However, other research has demonstrated that a carbon price has little effect on lowering carbon emissions (Klenert and Mattauch 2016; King et al. 2019).

## Model specification, data, and methodology

This paper aims to analyze the effect of environmental taxation, economic growth, renewable energy, and urbanization on CO_2_ emissions. The functional representation of this relationship is as follows:

1 $${CO}_{2}=f\left(GDP, {GDP}^{2}, TAX, REN, URB\right)$$

This nexus is expressed as a panel data model as follows:

2 $${CO2}_{it}={\alpha }_{it}+{\beta }_{1}{GDP}_{it}+{\beta }_{2}{GDP2}_{it}+{\beta }_{3}{TAX}_{it}+{\beta }_{4}{REN}_{it}+{\beta }_{5}{URB}_{it}+{\varepsilon }_{it}$$

where *i* implies each unit of the panel (EU-27 countries^1^), *t* denotes the data period (1995–2018). CO_2_ is the dependent variable and implies CO_2_ emissions in metric tons per capita. Independent variables are GDP per capita (constant 2015 US$), squared of GDP, environmental tax revenues (million dollars), the percentage of urban to total population, and the fraction of energy from renewable sources utilized for total final consumption of energy, respectively. The World Bank’s World Development Indicators provides other statistics, while the Eurostat database is the source of data for the environmental tax indicator. For every variable, the logarithmic transformation is utilized. Table 1 displays descriptive data for the series.

**Table 1:** Descriptive statistics

Variables	Obs	Mean	Max	Min	Skewness	Kurtosis	Std. dev
CO2	648	0.854	1.409	0.466	0.262	3.081	0.178
GDP	648	4.328	5.023	3.542	− 0.227	2.382	0.318
GDP^2^	648	18.836	25.231	12.546	− 0.079	2.363	2.732
TAX	648	3.542	4.901	1.514	− 0.112	2.350	0.722
REN	648	1.026	1.723	− 1.059	− 1.233	5.741	0.437
URB	648	1.847	1.991	1.704	− 0.031	2.102	0.075

In this study, the panel vector autoregression (PVAR) method is adopted to estimate Eq. (2). Before estimating the equation, whether the variables contain a unit root is checked by both IPS test developed by Im et al. (2003) and CIPS panel unit root test developed by Pesaran (2007). The reason why these tests are preferred is that the IPS considers the heterogeneity of the panel and the CIPS test handled also the cross-section dependence. After the unit root testing, the procedure for the PVAR approach is followed.

The PVAR approach is developed by Abrigo and Love (2016). The estimation procedure of this method is based on the Generalized Method of Moments (GMM) approach. This method provides a detailed empirical evidence framework by enabling long-run coefficient estimation, causality analysis, variance decomposition analysis, and obtaining impulse-response graphs for the relationship under consideration. The main PVAR model is constructed as follows:

3 $${Y}_{pt}=\sum \nolimits_{i=1}^{T}{A}_{i}{X}_{pt-i}+{\cup }_{c}+{\mu }_{ct}$$

where ${X}_{pt}=\left[{CO2}_{pt},{GDP}_{pt}{,GDP2}_{pt},{TAX}_{pt},{REN}_{pt},{URB}_{pt}\right]$ implies a vector of the exogenous variables. ${Y}_{pt}$ is $\left(kxk\right)$ vector of independent variables. ${\cup }_{c}$ is a vector of country-fixed effects and ${\mu }_{ct}$ is idiosyncratic error.

The PVAR approach considers unobserved heterogeneity and eliminates estimation errors caused by cross-sectional dependence panel VAR models add the cross-section to regular VAR models, but they are otherwise identical to standard VAR models in that each of the variables is endogenous and interdependent. The panel VAR method, however, has a few unique characteristics.

Due to the consideration of all endogenous variable delays, there is a dynamic interdependency between the variables. Additionally, error terms are typically associated across units; this characteristic is known as static interdependency.

Lastly, the shocks’ intercept, slope, and variance could vary depending on the unit. According to Canova and Ciccarelli (2013), this suggests that cross-sectional heterogeneity is available.

## Empirical findings

In the first stage of the analysis, it is tested whether the series are stationary or not. Regarding this, both IPS and CIPS test results are presented in Table 2. According to the IPS test results, it is understood that all variables are stationary at the first difference. When the results of the CIPS unit root test, which is another unit root testing approach adopted in this study, are examined, it is seen that the *GDP*, *GDP*^*2*^, and *TAX* variables are stationary at level, but others are stationary at first difference.

**Table 2:** Unit root test results

	IPS		CIPS	
Var	Level	First difference	Level	First difference
CO2	2.897	− 15.404^***^	− 1.812	− 5.406^***^
GDP	− 0.876	− 9.896^***^	− 2.162^*^	− 4.653^***^
GDP^2^	− 0.335	− 10.019^***^	− 2.101^*^	− 4.724^***^
TAX	− 0.672	− 13.204^***^	− 2.258^**^	− 5.734^***^
REN	− 3.554	− 15.901^***^	− 1.946	− 5.330^***^
URB	1.849	− 1.621^**^	− 1.310	− 2.272^**^

*, **, and *** denote 10%, 5%, and 1% statistically significance level, respectively

After the unit root tests, the panel VAR procedure is followed. First, the optimal lag length is determined, and the results are presented in Table 3. According to Table 3, it is concluded that the optimal lag length is 1 since the MBIC, MAIC, and MQIC have the lowest values at the lag(1).

**Table 3:** Optimal lag length selection

Lag	CD	*J*	*J*-value	MBIC	MAIC	MQIC
1	1	184.458	6.466	− 489.491	− 31.541	− 211.042
2	1	116.194	0.000	− 333.105	− 27.805	− 147.473
3	0.921	49.996	0.060	− 174.653	− 22.003	− 81.837

The long-run coefficient estimates determined using lag(1) are shown in Table 4. Because GDP has a positive influence on CO2 and GDP^2^ has a negative impact on it, initial data show the presence of an inverted U-shaped link between economic growth and air pollution, supporting the validity of the EKC hypothesis in these nations. Another finding indicates that environmental fees improve the quality of the air in these nations. An increase in environmental taxes reduces CO_2_ emissions by 0.14% in the European Union. This result is in line with Miller and Vela (2013), Guo and Wang (2018), Wolde-Rufael and Mulat-Weldemeskel (2022), Safi et al. (2021), Xu et al. (2023), and Nadiri et al. (2024). The findings regarding the relationship between environmental taxes and emissions may be evaluated in line with the widespread literature. This result confirms that the positive effects of environmental practices supported by strict policies are inevitable in the long run. On the other hand, the findings of Dmytrenko et al. (2024). Their results are important because they involve the comparison of two samples, central and western Europe. Since this study covers mostly Western European countries, it is compatible with their results for Western Europe. In fact, when the relevant literature and the results of this study are evaluated together, the success in the implementation of environmental taxes may be evaluated in connection with the institutional structure of the county or region and its success in public policies. Also, renewable energy consumption and urbanization have a negative impact on emissions, but this effect of renewable energy is statistically insignificant.

**Table 4:** Panel VAR long run coefficient estimation results

	CO2	GDP	GDP^2^	TAX	REN	URB
L.CO2	0.059	0.037^*^	0.288	1.068^**^	− 0.214^**^	− 0.000
L.GDP	9.486^***^	1.644^***^	16.795^***^	− 33.917^***^	− 4.545^***^	0.189^***^
L.GDP2	− 1.097^***^	− 0.187^***^	− 1.910^***^	3.835^***^	0.518^***^	− 0.021^***^
L.TAX	− 0.143^***^	− 0.025^***^	− 0.241^***^	0.258^***^	0.089^***^	− 0.002^***^
L.REN	− 0.035	− 0.012	− 0.122^*^	0.587^***^	− 0.001	− 0.000
L.URB	− 0.475^**^	− 0.168	− 1.883^***^	8.084^**^	− 0.091	− 0016^**^

*, **, and *** denote 10%, 5%, and 1% statistically significance level, respectively

Empirical results show that environmental taxes are an effective policy tool in tackling the problem of negative environmental externalities in European countries. The main purpose of environmental taxes is to reduce pollution by preventing activities that are harmful to the environment. Therefore, the results reflect a taxation system that serves this purpose. Beyond this direct effect, another measure of the effectiveness of environmental taxes is that these taxes encourage companies to develop environmentally friendly technologies and consume renewable energy. In these countries, the positive effect of environmental tax on renewable energy consumption is another important finding, and the effectiveness of environmental tax becomes clearer at this point. Accordingly, an increase in environmental tax revenues increases renewable energy consumption by about 0.09% in the long run. Therefore, this result means that tax revenues are used for sustainable purposes. Unlike the positive contribution of environmental tax to air quality and renewable energy consumption, it is observed that it reduces economic growth, albeit slightly. Its negative effect on economic growth is that taxes are an element that increases the cost of companies and therefore reduces international competition.

According to other empirical findings, both renewable energy consumption and urbanization cause an increase in environmental tax revenues. An increase in renewable energy consumption increases environmental tax revenues by about 0.6%, while an increase in urbanization increases it by about 8%. It is an expected conclusion that environmental tax revenues increase with urbanization. It can be said that urbanization accelerates industrialization and accordingly production and consumption process and causes environmental sanctions such as taxes to become widespread in case they pose a threat to environmental quality. At this point, it is possible to explain the reducing effect of urbanization on emissions. Therefore, environmental tax practices caused by urbanization mean that tax revenues increase and these revenues are qualified as a source for environmentally friendly incentive practices. The fact that renewable energy consumption causes an increase in environmental tax revenues reflects the deviations from the environmentally friendly approach to renewable energy consumption.

**Fig. 1:**
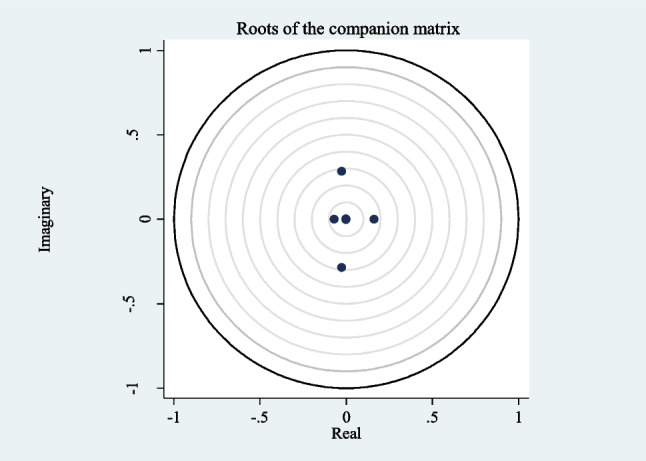
Stability of the PVAR model

Investigating the stability of the model discussed in the study is another stage of panel VAR analysis. The results of the stability test are presented both in Table 5 and graphically in Fig. 1. The fact that all the results in Table 5 are less than 1 and therefore all the points in Fig. 1 are within the unit circle indicates that the model is stable. This result allows the analysis to be considered in more detail and the causality test to be performed in the next step. Causality test results are reported in Table 6.

**Table 5:** Eigenvalue stability condition

Eigenvalue		Modulus
Real	Imaginary	
− 0.027	− 0.285	0.286
− 0.027	0.285	0.286
0.160	0	0.160
− 0.069	0	0.069
− 0.001	0.002	0.002
− 0.001	− 0.002	0.002

**Table 6:** Granger causality test results

	CO2	GDP	GDP^2^	TAX	REN	URB
CO2		3.513^*^	2.394	5.643^**^	5.992^**^	0.255
GDP	72.915^***^		93.384^***^	77.762^***^	44.850^***^	99.814^***^
GDP2	70.849^***^	84.131^***^		73.937^***^	40.656^***^	94.814^***^
TAX	68.265^***^	30.674^***^	36.862^***^		37.770^***^	55.491^***^
REN	0.794	2.382	2.895^*^	18.104^***^		1.939
URB	4.631^**^	12.451^***^	10.877^***^	5.205^**^	0.213	

*, **, and *** denote 10%, 5%, and 1% statistically significance level, respectively

The results of the causality test performed after the coefficient estimation point to some important findings. Accordingly, GDP and energy from renewable sources use have a one-way causal connection, as do emissions of carbon dioxide and renewable consumption of energy. Furthermore, bidirectional causation is shown between environmental tax and renewable energy use and urbanization, GDP and environmental tax and CO2 emissions, and so on. The causality test results support the interrelationships in the long-run coefficient estimation findings. Therefore, the strong links between emissions, environmental taxes, growth, clean energy consumption, and urbanization are emphasized once again. With this determination, in the next step, the variance decomposition between emissions and environmental tax is examined in the context of the main focus of the subject. The results obtained provide a significant inference.

Findings related to variance decomposition analysis are reported in Table 7 within the scope of emissions and environmental tax nexus. The first part of the table describes the change in emissions over 10 periods ahead through shocks in emissions and environmental tax. Accordingly, about 87% of changes in emissions are due to shocks in itself, while about 10% is caused by shocks in environmental taxes. In the second part of the table, changes in environmental taxes are explained by shocks in emissions and environmental tax. This part differs from the result in the first part of the table. Accordingly, the changes in environmental taxes over 10 periods ahead are explained by shocks in emissions of about 38%, while about 34% are explained by shocks in itself. Therefore, emissions have a critical role in the future of EU member countries as an important component that brings up the regulations regarding environmental tax.

**Table 7:** Variance decomposition analysis results

CO2 (response)	Impulse		TAX (response)	Impulse	
Forecast horizon (years)	CO2	TAX	Forecast horizon (years)	CO2	TAX
0	0	0	**0**	0	0
1	1	0	**1**	0.395	0.366
2	0.885	0.106	**2**	0.382	0.340
3	0.874	0.107	**3**	0.381	0.343
4	0.874	0.107	**4**	0.381	0.343
5	0.874	0.107	**5**	0.381	0.343
6	0.874	0.107	**6**	0.381	0.343
7	0.874	0.107	**7**	0.381	0.343
8	0.874	0.107	**8**	0.381	0.343
9	0.874	0.107	**9**	0.381	0.343
10	0.874	0.107	**10**	0.381	0.343

**Fig. 2:**
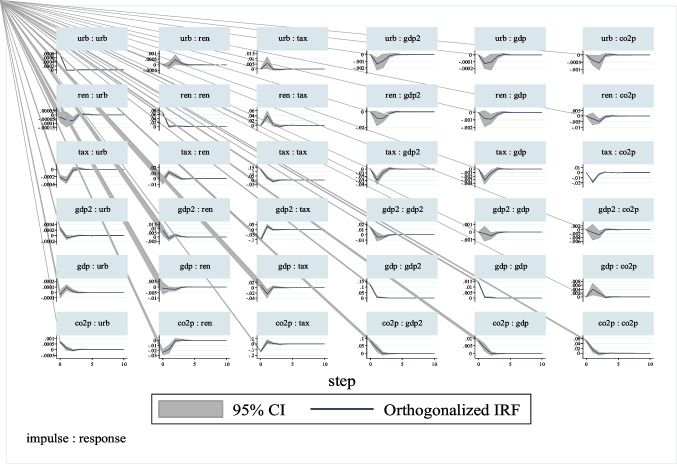
Impulse-response graphs

The shocks and their effects in the variance decomposition analysis can be represented more comprehensively and clearly in the impulse-response functions. These functions with 95% confidence intervals are presented graphically in Fig. 2. According to these results, the response of CO_2_ emissions to a standard deviation shock in GDP is firstly positive and then negative. However, the response of emissions to a standard deviation shock in urbanization, renewable energy consumption, and environmental tax is firstly negative, and then positive.

## Conclusion

This study examined the impact of economic development, urbanization, renewable energy usage, and environmental taxes on CO2 emissions in the EU-27. The PVAR technique was applied for this purpose between 1995 and 2018. The primary findings showed that urbanization, renewable energy use, and environmental taxes all had long-term detrimental effects on emissions. Additionally, the findings supported the EKC hypothesis’s validity in these nations.

In the light of the main findings, it is possible to make some policy implications for these countries. The results revealed that environmental taxes have a positive contribution to air quality more than renewable energy in these countries. However, considering that the growth-reducing effect of environmental taxes is greater than that of renewable energy consumption, the possible costs of environmental taxes should be reconsidered. At this point, it is necessary to complete the taxes applied for polluting sectors with a system that encourages the use of environmentally friendly technology to balance the costs. This means the development of a new reward-punishment approach in environmental regulations. One of the most critical points about taxes is the determination of tax rates. Accordingly, another important criterion is to determine the rates in polluting sectors by considering the sector-specific cost structures. With such an approach, a tax burden will be created that allows companies operating in related sectors to invest in environmentally friendly technologies. In these economies that are on the edge of economic, social, and democratic development, there is a suitable basis for more effective implementation of environmental taxes. Unless inactive choices (such as poverty) are made in the use of resources, developments such as environmental regulations in these countries produce positive results in favor of improving air quality.

In the following studies, factors such as economic growth, foreign trade, investment, poverty, democracy, corruption, and population can be considered the impact of environmental taxes on the effectiveness of environmental taxes, and the indirect effects of related factors on air quality, and how environmental taxes form the basis for the effectiveness of environmental taxes in developed and developing economies.

## Data Availability

The data used to support the findings of this study are included within the article.

## Footnotes

1 Austria, Belgium, Bulgaria, Croatia, Cyprus, Czech Republic, Denmark, Estonia, Finland, France, Germany, Greece, Hungary, Ireland, Italy, Latvia, Lithuania, Luxembourg, Malta, Netherlands, Poland, Portugal, Romania, Slovak Republic, Slovenia, Spain, Sweden.
